# Cancer metabolism: from the Warburg effect to precision therapy

**DOI:** 10.3389/fimmu.2026.1793553

**Published:** 2026-05-11

**Authors:** Canxuan Wang, Jing Wang, Lele Miao, Li Wei, Xinyuan Liu, Yan Lu, Longquan Xiang, Miaomiao Zhang

**Affiliations:** 1 Department of Clinical Medicine, Jining Medical University, Jining, China; 2 Department of Pathology, Jining First People’s Hospital, Jining, China; 3 Department of Thyroid and Breast Surgery, Jining No.1 People’s Hospital, Shandong, Jining, China; 4 Department of Medical Laboratory, Jining No. 1 People’s Hospital, Shandong First Medical University, Jining, China

**Keywords:** clinical application, metabolic reprogramming, targeted therapy, tumor metabolism, tumor micro-environment

## Abstract

Tumor cells undergo metabolic reprogramming to enable proliferation, survival, and metastasis, making tumor metabolism a key target in cancer research. This study examines current breakthroughs in metabolic reprogramming, including the metabolism of glucose, glutamine, fatty acids, and other nutrients. It describes how these metabolic changes affect anti-tumor immunity and the tumor microenvironment. The molecular processes of metabolic control are investigated. Furthermore, the review discusses practical applications resulting from this study, such as metabolism-based therapy techniques and diagnostic tools. Finally, it discusses future research objectives and difficulties, emphasizing the possibility of targeting tumor metabolism to improve precision cancer therapy.

## 1 Introduction

Cancer is one of the most serious diseases impacting worldwide human health. Its pathophysiology is complicated, involving various biological systems, and many researchers have conducted considerable and in-depth research on the subject (1). Tumor metabolism, an important subject in cancer research, has advanced significantly in recent years. Tumor cells have different metabolic patterns than normal cells during growth, proliferation, and metastasis (2). These metabolic changes not only provide tumor cells with plentiful energy and bio-synthetic precursors, but they also have an impact on the tumor microenvironment and host immunological responses, all of which play important roles in tumor start, development, and therapeutic response. Exploring tumor metabolism mechanisms not only broadens our fundamental understanding of cancer biology, but also provides new tactics and targets for early detection, precision therapy, and prognostic evaluation. This review systematically summarizes recent advances in tumor metabolism and their implications for therapy. It seeks to provide a solid theoretical foundation and reference framework for future research.

## 2 Characteristics of tumor cell metabolic reprogramming

### 2.1 Glucose metabolism

**Figure 1:**
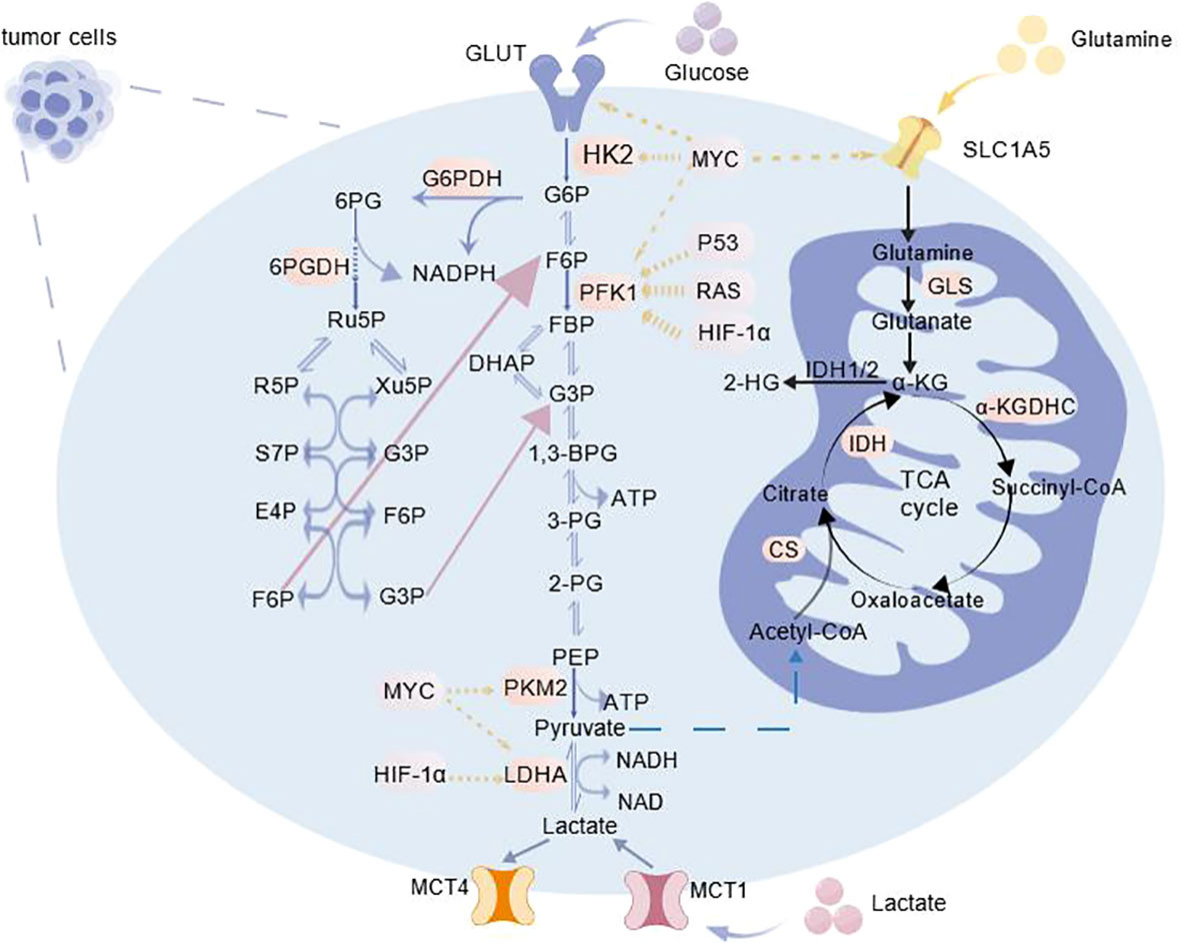
Glucose metabolism in tumor cells [Created with BioGDP.com (5)]. Tumor cells uptake glucose via elevated glucose transporters (GLUTs). Then it is converted to glucose-6-phosphate (G6P) by hexokinase 2 (HK2). G6P is metabolized to pyruvate via key enzymes including PFK1 and pyruvate kinase M2 (PKM2); pyruvate is then reduced to lactate by lactate dehydrogenase A (LDHA) and exported via monocarboxylate transporters 1/4 (MCT1/4). G6P also flows into the PPP to generate nicotinamide adenine dinucleotide phosphate(NADPH) and ribose-5-phosphate (R5P) for biosynthesis and antioxidant defense. Concurrently, glutamine is imported via solute carrier family 1 member 5(SLC1A5) and catabolized by glutaminase (GLS) to α-ketoglutarate (α-KG) for the TCA cycle. mutations in isocitrate dehydrogenase 1/2(IDH1/2) redirects α-KG to produce the oncometabolite 2-HG. These pathways coordinately sustain rapid proliferation, redox balance, nucleotide/lipid synthesis, and malignant progression via oncogenic signaling.

In the late 1930s, German scientist Otto Warburg undertook systematic study on tumor cell energy metabolism, establishing for the first time the unique metabolic phenotype that separates tumor cells from normal cells. This groundbreaking discovery, later called the Warburg effect (3, 4). It is distinguished by the following key feature: even under aerobic conditions, tumor cells preferentially metabolize glucose via glycolysis, resulting in significant lactic acid generation. The buildup of lactic acid alters the tumor microenvironment (**Figure 1**) (6, 7). Glycolysis does not rely on mitochondrial structures; rather, it is completed by a series of 10 enzymatic processes in the cytoplasm. This method is more faster than aerobic oxidation, which involves the coordinated action of numerous organelles, allowing tumor cells to receive instant energy. More critically, glycolysis produces several crucial precursors required for biosynthesis. For example, ribose-5-phosphate produced by the pentose phosphate route can be used directly for nucleotide synthesis, but 3-phosphoglycerate is a substrate for amino acid synthesis (8, 9). The efficient use of these metabolic intermediates allows tumor cells to strike a dynamic balance between energy supply and biosynthesis, laying the metabolic groundwork for malignant proliferation, invasion, metastasis, and other biological activities. However, glycolysis does have limitations. Glycolysis produces less ATP molecules. This means that cancer cells must consume more glucose to meet their high energy requirements, making them heavily reliant on glucose supply. If glucose availability is restricted, cancer cells’ growth and survival would be severely hampered (10). At the same time, the creation of certain intermediate metabolites may not be sufficient to meet tumor cells’ huge demands for infinite multiplication.

PFK1 plays a critical role in the glycolytic process, making its expression regulation mechanism an active study area.PFK1 catalyzes the major irreversible step and critical regulatory point in glycolysis: the phosphorylation of fructose-6-phosphate to fructose-1,6-bisphosphate (11, 12). This phase directly affects the efficiency of glucose metabolism. Enhanced activity dramatically enhances the rate of lactate synthesis in tumor cells, delivering enough ATP and intermediate metabolites for rapid tumor growth (13). Notably, glycolytic enzymes not only play a role in energy metabolism but also influence tumor spread via nonmetabolic routes. For example, enolase (ENO1), which catalyzes the glycolytic pathway’s conversion of 2-phosphoglycerate to phosphoenolpyruvate, can act as a plasminogen receptor on the cell membrane. This allows it to convert plasminogen into plasmin, which then breaks down extracellular matrix (ECM) components such type IV collagen and laminin. This process breaks down tissue barriers and encourages tumor cell invasion. Degrades the extracellular matrix (14). In addition, glycolytic enzymes such as phosphoglycerate kinase 1 (PGK1) and aldolase A (ALDOA) can promote the proliferation and invasive capacity of tumor cells (15, 16).

In addition to the classic Warburg effect, tumor cells can adapt to their proliferative needs through multiple glucose metabolic pathways; among these, the abnormal activation of the pentose phosphate pathway (PPP) is a key feature of tumor metabolic reprogramming and treatment resistance. Metabolomics and flux studies have confirmed that glucose-6-phosphate dehydrogenase (G6PDH), a key rate-limiting enzyme in the PPP, is significantly upregulated in various tumors, suggesting that the PPP is a central component driving metabolic remodeling (17). Overexpression of G6PD promotes tumorigenesis; in a KRAS-mutation-driven lung cancer model, knocking out G6PD significantly inhibits tumor growth, and this effect is independent of changes in reactive oxygen species (ROS) levels, implying that the PPP primarily drives tumor proliferation by promoting nucleotide synthesis via the provision of ribose-5-phosphate (R5P). The PI3K/AKT/mTOR pathway directly phosphorylates and activates G6PD, while the transcription factor MYC upregulates critical PPP genes like TKT and TALDO1, transforming the PPP into a downstream executor module of the oncogenic signaling network. This increases metabolic flux, supplying the reducing power (NADPH) and synthesis precursors required for fast tumor growth (18, 19). The PPP, which shares the substrate glucose-6-phosphate with glycolysis, works through the synergistic interaction of its oxidative and non-oxidative phases: the oxidative phase produces NADPH, maintains redox balance, and supports lipid and nucleic acid synthesis; the non-oxidative phase produces R5P and glycolytic intermediates, facilitating the supply of synthetic substrates and flexible integration with core metabolic pathways (20, 21). This metabolic feature allows the PPP pathway to independently supply tumor cells with raw materials while reducing metabolic power requirements, as well as flexibly integrate with core metabolic pathways to assist tumors in adapting to environmental changes (such as nutrient deprivation and oxidative stress) (22).

Metabolic conditions within tumors exhibit spatial heterogeneity. Significant gradients of oxygen and nutrients exist within solid tumors. For example, in hypoxic core regions, cells are forced to rely on glycolysis for energy; in oxygen-rich peripheral regions of the tumor, cells may be more inclined to undergo oxidative phosphorylation (OXPHOS).Recent research suggests that the tricarboxylic acid (TCA) cycle in tumor cells is not totally repressed, but rather “reprogrammed” to fulfill the bioenergetic and metabolic demands required for fast proliferation (23, 24). When oxygen is abundant, normal cells efficiently create significant amounts of ATP mostly via the TCA cycle and oxidative phosphorylation. In contrast, tumor cells convert more pyruvate into lactate even in aerobic settings, with just a tiny fraction of pyruvate entering the TCA cycle. As a result, they make relatively little ATP through oxidative phosphorylation (25). The TCA cycle in tumor cells frequently produces enough metabolic intermediates to sustain the production of macromolecules including nucleotides, amino acids, and lipids, fulfilling the demands of fast growth. Citrate, for example, is a TCA cycle intermediate that can be used for *de novo* fatty acid synthesis, namely the creation of cell membrane lipids, in order to sustain tumor cell proliferation (26). Second, genetic alterations in TCA cycle enzymes have been found in a variety of tumors, establishing a clear relationship between the TCA cycle and tumorigenesis and development. For example, mutations in isocitrate dehydrogenase (IDH), succinate dehydrogenase (SDH), and fumarate hydratase (FH) cause the buildup of certain metabolic intermediates such 2-hydroxyglutarate. These intermediates can induce cancer through many pathways, including epigenetic changes (27, 28).

There is a central debate regarding glycolysis and oxidative phosphorylation: Do tumor cells rely primarily on glycolysis? OXPHOS? Or do the two coexist dynamically? This is precisely regulated by a multi-layered regulatory network. First layer: The interplay between oncogenes and tumor suppressor genes. The oncogenes MYC and HIF-1α are key transcription factors driving glycolysis. Tumor-suppressor signals such as AMPK and p53 tend to promote OXPHOS and inhibit glycolysis. However, it remains unclear how these signals are integrated into a unified decision-making process. Second layer: Microenvironmental signals. Oxygen concentration gradients, nutrient supply, and pH levels in the tumor microenvironment directly influence the selection of metabolic phenotypes. Tumor cells in hypoxic regions rely more heavily on glycolysis, while those in oxygen-rich areas near blood vessels may utilize OXPHOS to a greater extent. Third layer: Post-translational modifications and regulation of metabolic enzymes. Post-translational modifications of metabolic enzymes, such as phosphorylation, acetylation, and ubiquitination, can rapidly alter enzyme activity, enabling immediate shifts in metabolic states.

### 2.2 Amino acid metabolism

Glutamine is another essential food for tumor cells, serving many functions in tumor metabolism. It functions as a critical metabolic center for tumor cell survival, proliferation, and drug resistance development by precisely regulating redox homeostasis and metabolic adaptability. Its metabolites can be used to synthesize biomolecules like nucleotides and amino acids (29). Under glucose metabolism-restricted conditions, glutamine is transformed into glutamate by glutaminase (GLS) activity. This glutamate is then converted into TCA cycle, which provides energy to tumor cells (**Figure 1**) (30).

Furthermore, tumor cells are not only glutamine-dependent, but they also absorb large amounts of glutamine from the microenvironment via upregulation of transporters such as ASCT2/SLC1A5 and LAT1/SLC7A5, resulting in local nutrient depletion and suppression of T-cell function, thereby mediating immune evasion (31, 32). Mutated IDH in tumors, such as glioblastoma, transforms α-KG into the carcinogenic metabolite 2-HG. This causes the citric acid cycle and antioxidant activities to rely heavily on glutamine-derived α-KG, increasing glutamine addiction. Thus, inhibiting glutaminase (GLS) can drastically reduce tumor cell growth and proliferation (33, 34).

Glutamine also helps tumor cells maintain their redox equilibrium. Glutaminase (GLS1/2) converts glutamine to glutamic acid, which is then further transformed into α-ketoglutarate (α-KG) by glutamate dehydrogenase (GDH) or transaminase. α-KG activates antioxidant enzymes, maintaining ROS-clearing proteins like catalase and superoxide dismutase, and minimizing oxidative damage (35). This technique maintains redox equilibrium in tumor cells during periods of rapid growth and metabolic stress, thereby increasing their antioxidant capacity (36).

Furthermore, ammonia generated from glutaminolysis can serve as a nitrogen source for the synthesis of purines and pyrimidines, thereby supporting the construction of DNA and RNA. DNA damage repair, including PARP1-mediated repair, requires a large amount of NAD^+^ as a substrate. At the same time, glutamate-derived acetyl-CoA can participate in lipid synthesis, maintain cell membrane integrity, and reduce drug-induced damage to membrane structures. Glutamine metabolism also exhibits cross-regulation with drug resistance-related signaling pathways, with the PI3K/AKT and Nrf2 pathways being particularly significant. The AKT signaling pathway activates GLS1 through phosphorylation, while Nrf2, as a key core transcription factor in antioxidant responses, directly binds to the promoter regions of GLS1 and glutamate-cysteine ligase catalytic subunit (GCLC), thereby promoting their expression. This forms a positive feedback loop that further enhances tumor cell drug resistance (37, 38).

Although tumor cells generally exhibit glutamine dependence, their metabolic plasticity allows them to effectively escape this characteristic under specific conditions, thereby sustaining proliferation and survival. For instance, when abundant carbon sources such as lactate and ketone bodies are present in the tumor microenvironment, tumor cells can also maintain metabolic demands using these substrates and overcome glutamine dependence. When glutamine is blocked, tumor cells activate various alternative anaplerotic pathways to sustain energy supply and metabolic demands. The reprogramming of the serine synthesis pathway (SSP) is critical. Studies have shown that prolonged glutamine deprivation or glutaminase (GLS) inhibition causes breast cancer cells to upregulate the essential SSP enzymes PHGDH, PSAT1, and PSPH via the AMPK signaling pathway. SSP primarily produces α-KG, which refills the TCA cycle through anaplerotic means. This function makes PSAT1 a crucial metabolic node when glutamine is blocked. Stromal cells can also protect against metabolic stress during glutamine deficiency. CAFs release aspartate, alanine, and even glutamine, which directly supplies amino acids to tumor cells. More crucially, CAFs can deliver critical metabolic enzymes like glutaminase 2 (GLS2) to tumor cells via exosomes, allowing them to generate or use glutamine locally. These compensatory mechanisms not only show the complexities of tumor metabolism, but also explain why glutamine-targeting monotherapies frequently have poor success in clinical settings.

In addition to glutamine, the metabolism of other amino acids like arginine and leucine is strongly linked to tumor cell development and proliferation. Arginine can produce nitric oxide (NO) via the nitric oxide synthase (NOS) pathway, which aids in tumor cell signaling and angiogenesis. Tumor cells compete with immune cells for arginine, which increases arginine levels within tumor cells and promotes tumor cell proliferation (39). It also affects T cell activity, which allows tumor cells to avoid the immune system. Methionine metabolism is closely linked to methylation changes in tumor cells. It contributes to the methylation of DNA, RNA, and proteins by providing the methyl donor Sadenosylmethionine (SAM), regulating gene expression and physiological functioning (40).

### 2.3 Fatty acid metabolism

**Figure 2:**
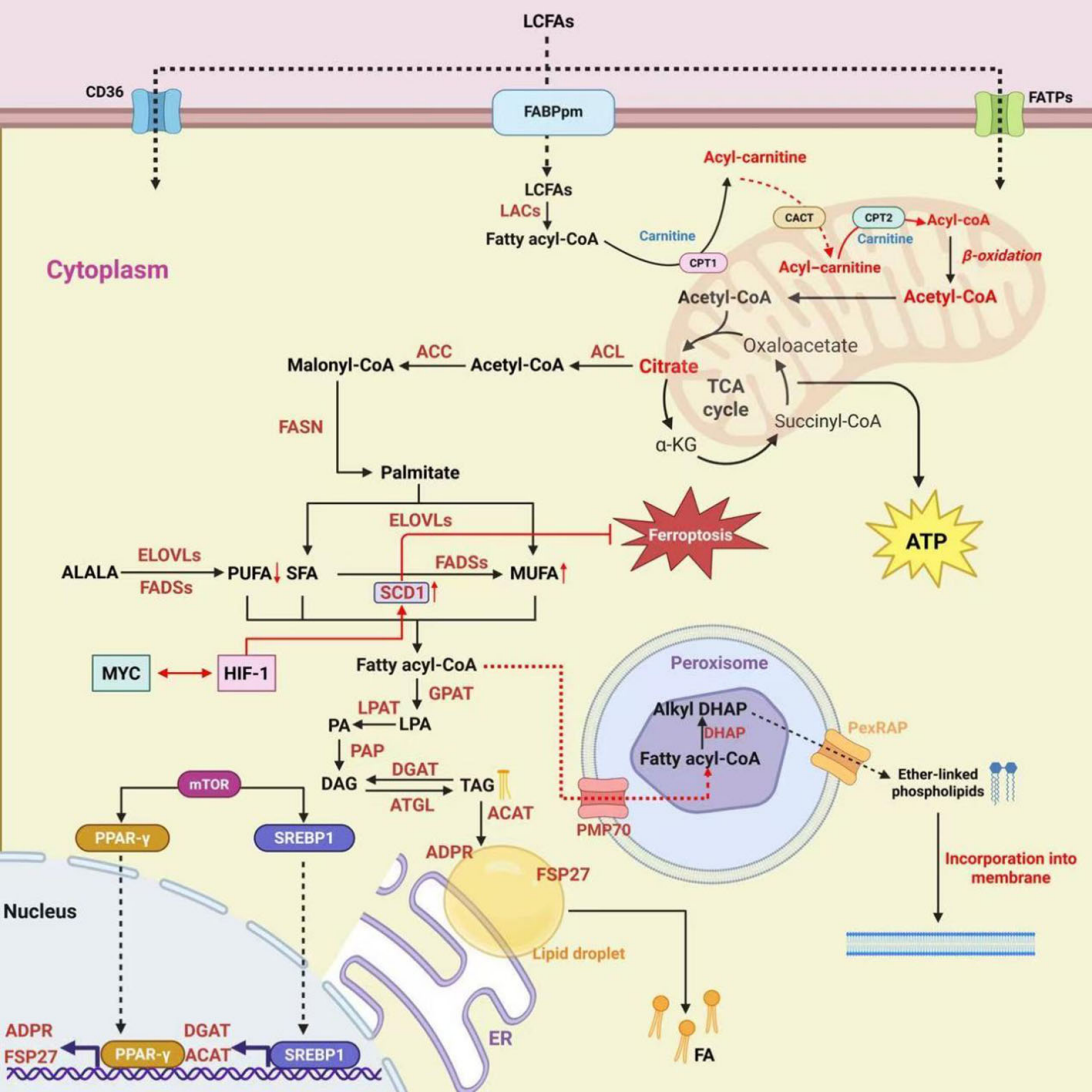
Fatty acid metabolism in tumor cells. Exogenous fatty acids are imported via CD36, fatty acid transport proteins (FATPs), and plasma membrane fatty acid-binding protein (FABPpm), then activated to fatty acyl-CoA. Acyl-CoA enters mitochondria via CPT1/CPT2 for β-oxidation and ATP production. Citrate-derived acetyl-CoA is converted by ATP citrate lyase(ACL),acetyl-CoA carboxylase(ACC), and FASN to palmitate, then modified by elongation of very long chain fatty acids(ELOVLs), fatty acid desaturases (FADSs), and stearoyl-CoA desaturase 1 (SCD1). Lipids are stored in lipid droplets (GPAT, DGAT, FSP27, ADPR). Polyunsaturated fatty acids(PUFA) accumulation triggers ferroptosis. These processes support membrane biogenesis, energy supply, ferroptosis resistance, and metastasis, and are transcriptionally controlled by mTOR, MYC, HIF-1, sterol regulatory element-binding protein 1(SREBP1), and peroxisome proliferator-activated receptor γ(PPAR-γ).

Fatty acid metabolism plays a crucial role in the proliferation, survival, and metastasis of tumor cells (41, 42). To address their metabolic needs, tumor cells can either absorb external fatty acids or manufacture fatty acids from scratch (**Figure 2**). In terms of fatty acid synthesis, tumor cells exhibit markedly elevated expression of fatty acid synthase (FASN), which stimulates the synthesis of fatty acids (43). ACL (ATP citrate lyase) catalyzes the conversion of citrate into acetyl-CoA, which is further carboxylated to malonyl-CoA by ACC (acetyl-CoA carboxylase). Malonyl-CoA is then used for the *de novo* synthesis of palmitate catalyzed by FASN. Subsequently, various fatty acids with different degrees of saturation are produced via ELOVLs (elongation of very long chain fatty acids), FADSs (fatty acid desaturases), and SCD1 (stearoyl-CoA desaturase 1) (44, 45). Additionally, tumor cells can also take up exogenous fatty acids by upregulating transporters such as CD36,FATPs (fatty acid transport proteins) and FABPpm (plasma membrane fatty acid-binding protein) (46). In breast cancer cells, dipeptidyl peptidase-3 expression is upregulated, which stabilizes high FASN expression and promotes tumor fatty acid synthesis (47). FASN plays a role in phospholipid production and tyrosine kinase receptor signaling in renal cancer cells. It is therefore anticipated that reducing FASN activity may induce tumor cell death, stop the cell cycle, and reduce tumor growth (48).

Tumor cells can utilize fatty acid oxidation (FAO) to generate energy (49). Tumor cells can stimulate the fatty acid oxidation pathway in response to food shortage or hypoxia, converting fatty acids into acetyl-CoA, which subsequently enters the TCA cycle to produce ATP (50). FAO is also connected with tumor cells’ metastatic potential. According to studies, in tumor cells with strong metastatic potential, the expression of FAO-related genes is elevated, increasing tumor cell motility and invasive capacity. FAO-driven metabolic reprogramming helps tumor cells adapt to the microenvironment at metastatic locations (51).

In addition, under the dynamic regulation of lipid droplet-associated proteins including FSP27 and ADPR, tumor cells can catalyze the production of triacylglycerol (TAG) from acyl-CoA via a series of enzymes such as glycerol-3-phosphate acyltransferase (GPAT) and diacylglycerol acyltransferase (DGAT), which is then stored in lipid droplets.

The tumor microenvironment is rich in fatty acids, which stimulates the growth and multiplication of M2 macrophages, a subgroup that aids tumor cell growth (52). M2 macrophages secrete VEGF and other substances to stimulate angiogenesis in tumor cells, which provides nutrition and oxygen to the tumor. Simultaneously, they produce matrix metalloproteinases (MMPs), which damage the extracellular matrix, allowing tumor cells to penetrate tissue barriers and promote invasion and metastasis. As a result, limiting fatty acid buildup in tumor-associated macrophages can prevent tumor growth (53).

Phospholipids are critical components of the cell membrane’s bilayer structure, controlling fluidity, permeability, and signal transduction capabilities (54). Fatty acids act as precursors for phospholipids. Tumor cells guarantee an appropriate supply of phospholipids for the formation of new cell membranes by controlling fatty acid production and absorption, fulfilling the needs of their rapid division (55). Tumor cells regulate membrane fluidity by adjusting the ratio of monounsaturated to polyunsaturated fatty acids. An increase in monounsaturated fatty acids (MUFA) improves membrane stability and reduces susceptibility to lipid peroxidation, preventing ferroptosis. This promotes the development of drug resistance in tumor cells (56).

Furthermore, FAO increases tumor cell resistance to chemotherapeutic treatments by influencing mitochondrial activity and signaling pathways like STAT3. Inhibiting FAO enhances the sensitivity of tumor cells to chemotherapy (57). FAO occurs largely within mitochondria. Tumor cells increase the transport and oxidation of fatty acids into mitochondria by activating FAO-associated enzymes. For example, ACSL enzymes impact cell membrane phospholipid composition by catalyzing particular PUFA esterification, hence modulating ferroptosis sensitivity. Tumor cells may alter mitochondrial functional states by selectively using specific fatty acids or altering ACSL activity, so avoiding the toxicity of chemotherapeutic medicines. The STAT3 signaling pathway is strongly linked to tumor cell proliferation, survival, and immune evasion. FAO products or metabolic intermediates may alter STAT3 phosphorylation and nuclear translocation, increasing transcriptional activity. Activated STAT3 can increase the expression of anti-apoptotic genes such Bcl-2, lowering tumor cell sensitivity to chemotherapy treatments (58).

### 2.4 Other metabolites

In tumor metabolic reprogramming research, the essential functions of glucose, amino acids, and fatty acids have attracted a lot of attention. To address complicated needs such as fast proliferation, microenvironment adaptability, and immune evasion, tumor cells’ metabolic networks are significantly more complex than these three classical substrates. Numerous non-canonical metabolic pathways, such as nucleotide metabolism and metal ion metabolism, play critical roles in tumor growth but have only recently emerged as new study focus areas (59).

Nucleotide metabolism, as a key route in cell proliferation, not only provides the raw materials for DNA replication and RNA transcription in tumor cells, but it is also intimately related to the tumor microenvironment and immune function. Tumor cells generally acquire nucleotides via two pathways: salvage synthesis and *de novo* synthesis. Tumor cells preferentially activate *de novo* synthesis pathways to meet their significant nucleotide demands, as evidenced by significantly increased expression and activity of multiple key enzymes within this pathway (60). For example, the oncogene Ras regulates the expression of phosphoribosyl pyrophosphate (PRPP) synthase (PRPS1), the initiating enzyme for *de novo* synthesis (61). Its expression levels in colorectal and liver cancer tissues are 2–3 times greater than in normal tissues, which speeds up purine nucleotide synthesis via increasing PRPP production (62, 63). Furthermore, the *MYC* transcription factor directly activates dihydrofolate reductase (DHFR) and thymidylate synthase (TYMS), the rate-limiting enzymes in *de novo* pyrimidine nucleotide synthesis in lung and breast malignancies. Their overexpression is strongly linked to tumor cell resistance to chemotherapeutic treatments (64).

Monocarbon metabolism (MCM) generates one-carbon units for new nucleotide synthesis. It is mostly made up of the folate and methionine cycles, which are often quite active in tumor cells (65). In the cytoplasm, serine hydroxymethyltransferase (SHMT) breaks down serine to create glycine and 5,10-methylene tetrahydrofolate. 5,10-Methylene Tetrahydrofolate (5,10-CH_2_-THF) is a key one-carbon unit donor. The methionine cycle is largely involved in the synthesis and regeneration of S-adenosylmethionine (SAM). SAM is the primary intracellular methyl donor, involved in the methylation of DNA, RNA, and proteins, which is critical for gene expression regulation and epigenetic changes (66). The one-carbon route also contributes to the production of reducing cofactors like NADPH, which enable tumor cells maintain redox balance and fight cell death (67). Given the importance of one-carbon metabolism in carcinogenesis and progression, it has emerged as a critical target for cancer detection and treatment. For decades, antifolate medicines that inhibit one-carbon metabolism, such as methotrexate, have been frequently used in cancer treatment.

Iron metabolism in tumor cells exhibits systematic reprogramming distinct from that of normal cells, in order to meet the metabolic demands of unlimited proliferation, migration, and adaptation to oxidative stress. This reprogramming is primarily manifested as a coordinated dysregulation across four key processes: iron uptake, storage, utilization, and efflux. First, tumor cells typically overexpress transferrin receptor 1 (TFR1) to enhance the uptake of iron bound to transferrin, ensuring an adequate iron supply to support critical biochemical processes such as iron-sulfur cluster and heme synthesis. Second, upregulation of ferritin expression is a common feature of tumor cells. This not only provides a reserve iron source for proliferation-induced stress but also mitigates oxidative damage caused by the iron-dependent Fenton reaction by reducing intracellular free iron (i.e., the labile iron pool).At the same time, most tumor cells downregulate the expression of the membrane iron transporter ferroportin (FPN), reducing iron efflux to maintain intracellular iron retention. This mechanism not only supports proliferation-promoting pathways such as mitochondrial respiration and nucleotide synthesis but also reshapes the sensitivity of tumor cells to ferroptosis (68). Furthermore, different tumors exhibit significant differences in lipid metabolism, iron metabolism, and sensitivity to ferroptosis. These characteristics are shaped by both tumor-intrinsic properties and the microenvironment, thereby determining their varying responses to metabolic interventions. For example, melanoma relies on MUFA provided by the lymphatic microenvironment and establishes the strongest barrier against ferroptosis through iron homeostasis regulation mediated by the mevalonate pathway and SREBP2; therapeutic strategies for this cancer focus on lipid uptake nodes such as CD36 and ACSL3. Breast cancer, on the other hand, exhibits significant subtype heterogeneity. Although triple-negative breast cancer (TNBC) has high iron accumulation, it maintains resistance to ferroptosis by enhancing glutathione synthesis and downregulating ACSL4 through the TGF-β1/GGT1 axis. metabolic interventions for TNBC require a combined approach targeting tumor-associated macrophages (TAMs) and key enzymes of lipid synthesis (such as FASN and SCD1). Ovarian cancer is characterized by lipid uptake driven by lysosomal phosphatidylcholine secreted by CAFs; although free iron levels are high, it resists lipid peroxidation through the storage of polyunsaturated fatty acids (PUFAs) in lipid droplets and SELENOI-mediated ACSL4 inhibition, making it more sensitive to strategies that jointly target FABP4 and glutamine metabolism (69). In summary, the microenvironment-driven metabolic reprogramming of these three subtypes constitutes the core difference in their sensitivity to ferroptosis and provides a theoretical basis for precision therapies targeting metabolic heterogeneity.

Copper metabolism, a critical branch of metal ion metabolism, has been shown in recent years to control tumor cell activity and the tumor microenvironment via numerous routes. Under physiological settings, copper acts as a trace element, participating in redox reactions, enzyme activity regulation, and other processes. Vascular endothelial cells and CAFs produce copper ions in the tumor microenvironment, resulting in a 1.5 to 4-fold increase in copper content in tumor tissues compared to normal tissues. Its regulation mechanism manifests primarily in three areas: Copper can activate the NF-κB signaling pathway, boosting PD-L1 transcription and membrane location. This allows tumor cells to avoid immune monitoring by CD8+ T cells (70, 71). Second, copper, as a key cofactor of mitochondrial respiratory chain complex IV, maintains its catalytic activity via binding to MT-CO1 and MT-CO2. This ensures the effectiveness of mitochondrial oxidative phosphorylation (OXPHOS), which supplies tumor cells with adequate ATP (72). Third, copper promotes autophagy by influencing the dynamic equilibrium of autophagy-related proteins ULK1/ULK2 and CRIP2 (73, 74). When intracellular copper concentrations rise, copper ions bind to CRIP2 and block its function, loosening CRIP2’s control over ULK1/ULK2. This stimulates autophagosome production, such as nutrition deprivation, hence increasing their survival advantage (75, 76). Interestingly, high copper causes a distinct type of cell death known as copper death (77). Copper can catalyze the development of reactive oxygen species (ROS), resulting in oxidative stress, which destroys tumor cell components and triggers apoptosis. Excess copper can also directly or indirectly alter the activity of apoptosis-related proteins, such as *p53*, which promotes tumor cell death (78).

## 3 Molecular mechanisms of tumor metabolic reprogramming

According to modern oncology perspectives, metabolic reprogramming is directly linked to cancer hallmarks such as continuous proliferative signals and genetic abnormalities (79). Mutations in driver genes and signaling pathway dysregulation bypass normal cellular metabolic checkpoints, directing cells toward a state that promotes fast biosynthesis and cell proliferation (80). As a result, studying the molecular processes that regulate tumor metabolism is critical not only for understanding carcinogenesis and development, but also for creating precision medicines that target metabolic pathways (81).

### 3.1 Direct transcriptional and enzymatic regulation of tumor metabolism by core driver genes

Driver genes in tumor metabolic control are those that, by genetic or epigenetic changes, directly influence tumor cell metabolic pathways, driving tumor genesis, development, and maintenance. Oncogenes and tumor suppressor genes are typical examples of genes that adapt tumor cells to the harsh tumor microenvironment and encourage aberrant proliferation by influencing the expression and activity of metabolic enzymes, signaling pathways, and cellular nutrient utilization ability.

*MYC* is an important tumor suppressor gene that acts as a broad-spectrum transcription regulator and is overexpressed in over half of malignancies (82). It controls entire metabolic networks by attaching directly to the promoter or enhancer regions of multiple metabolic genes (83). *MYC* directly regulates the expression of the glucose transporter GLUT1 and the majority of glycolytic enzymes, hence powerfully promoting glycolysis (84). Among these, the induction of PKM2 is particularly crucial (85). Reduced PKM2 enzyme activity causes the buildup of glycolytic intermediates, which serve as substrates for branch pathways like the pentose phosphate pathway and serine production. *MYC* also stimulates glutaminase expression, which promotes glutamine uptake and breakdown by cancer cells (86). This mechanism replenishes α-ketoglutarate in the tricarboxylic acid cycle and supplies nitrogen for nucleotide and amino acid synthesis (87). Furthermore, it contributes to the synthesis of glutathione, the most important antioxidant. *MYC* also controls lipid synthesis (via SREBP), mitochondrial biogenesis, and miRNA expression, resulting in a comprehensive anabolic program (88, 89).

*p53*, as a crucial tumor suppressor, plays a central role in tumor metabolic reprogramming. Functional *p53* inhibits glycolysis, boosts oxidative phosphorylation, and increases glutamine flow into the tricarboxylic acid cycle. Inactivation of *p53* eliminates glycolytic inhibition, increases glucose utilization, and reduces ROS production, giving tumor cells a survival advantage (90). Wild-type *p53* also regulates critical enzymes in the glutamine metabolic pathway, such as decreasing GLS expression, which reduces glutamine conversion to glutamate and hence inhibits tumor cell proliferation. Mutant *p53* can upregulate glutamine transporters, increasing glutamine uptake in tumor cells, while also increasing GLS expression to satisfy their high metabolic demands. Mutant *p53* in cancers regulates fatty acid metabolism in more sophisticated ways. Its impacts go beyond the loss of wild-type *p53*’s tumor-suppressing role; it also promotes tumor cell metabolic reprogramming via gain-of-function (GOF) processes, which influences tumor initiation, progression, invasion, and treatment resistance.

### 3.2 Network-based integrated regulation of tumor metabolism by core signaling pathways

The PI3K-AKT-mTOR pathway is the primary mechanism that stimulates anabolic metabolism in cells when they receive growth factor signals (91, 92). This system combines many external and intracellular inputs to control a variety of cellular functions, including growth, proliferation, and metabolism. Its dysregulation is frequently reported in a variety of clinical conditions, including cancer and metabolic illnesses.

Growth factor receptors (EGFR, IGF-1R) are normally activated to launch the PI3K-AKT-mTOR pathway. Once activated, these receptors recruit and activate phosphoinositide 3-kinase (PI3K). Activated PI3K catalyzes the phosphorylation of phosphatidylinositol 4,5-bisphosphate (PIP2) into phosphatidylinositol 3,4,5-trisphosphate. PIP3 acts as a second messenger, recruiting AKT (also known as protein kinase B) and phosphoinositide-dependent kinase 1 (PDK1) to the cell membrane. AKT is the pathway’s fundamental component; once activated, it phosphorylates a number of downstream proteins, the most important of which is mammalian target of rapamycin (mTOR). mTOR exists in two different multiprotein complexes: mTORC1 and mTORC2. Specifically, mTORC1 governs protein synthesis and lipogenesis, whereas mTORC2 controls AKT phosphorylation and activation, as well as cytoskeleton rearrangement.

In glucose metabolism, AKT stimulates glucose absorption by promoting GLUT1/4 membrane translocation. For example, insulin signaling via the PI3K-AKT pathway causes the transfer of GLUT4 from intracellular vesicles to the plasma membrane, boosting glucose absorption in muscle and adipose tissues (93). Furthermore, studies show that oncogenic activation of the PI3K-AKT pathway increases GLUT1 localization to the plasma membrane, which promotes glucose utilization in cancer cells (94).

In terms of protein metabolism, mTORC1 upregulates the solute carrier (SLC) family, thereby accelerating the uptake of amino acids such as glutamine and serine by tumor cells; meanwhile, AKT activates GLS1/2, which hydrolyzes glutamine to glutamate that is further converted to α-HG for entry into TCA cycle. AKT activates mTORC1 by inhibiting the TSC1/TSC2 complex (95). The TSC1/TSC2 complex works as a GTPase-activating protein (GAP) for the brain-enriched Ras homolog protein (Rheb), keeping it inactive and GDP-bound. When activated, AKT phosphorylates TSC1/TSC2, which inhibits its GAP action. This permits Rheb to accumulate in an active GTP-bound state, thereby activating mTORC1. mTORC1 then phosphorylates two critical downstream target proteins, 4E-BP1 and S6K1 (96). When phosphorylated, 4E-BP1 dissociates from eIF4E, allowing eIF4E to start cap-dependent mRNA translation (97). Simultaneously, active S6K1 phosphorylates ribosomal protein S6, boosting ribosomal biosynthesis and driving the translation of mRNAs that encode ribosomal proteins and other growth-related proteins. This dramatically improves protein translation start and ribosome synthesis (98, 99). The aforementioned process results in a considerable increase in protein synthesis, which is the critical anabolic process required for tumor cell growth and proliferation.

**Figure 3:**
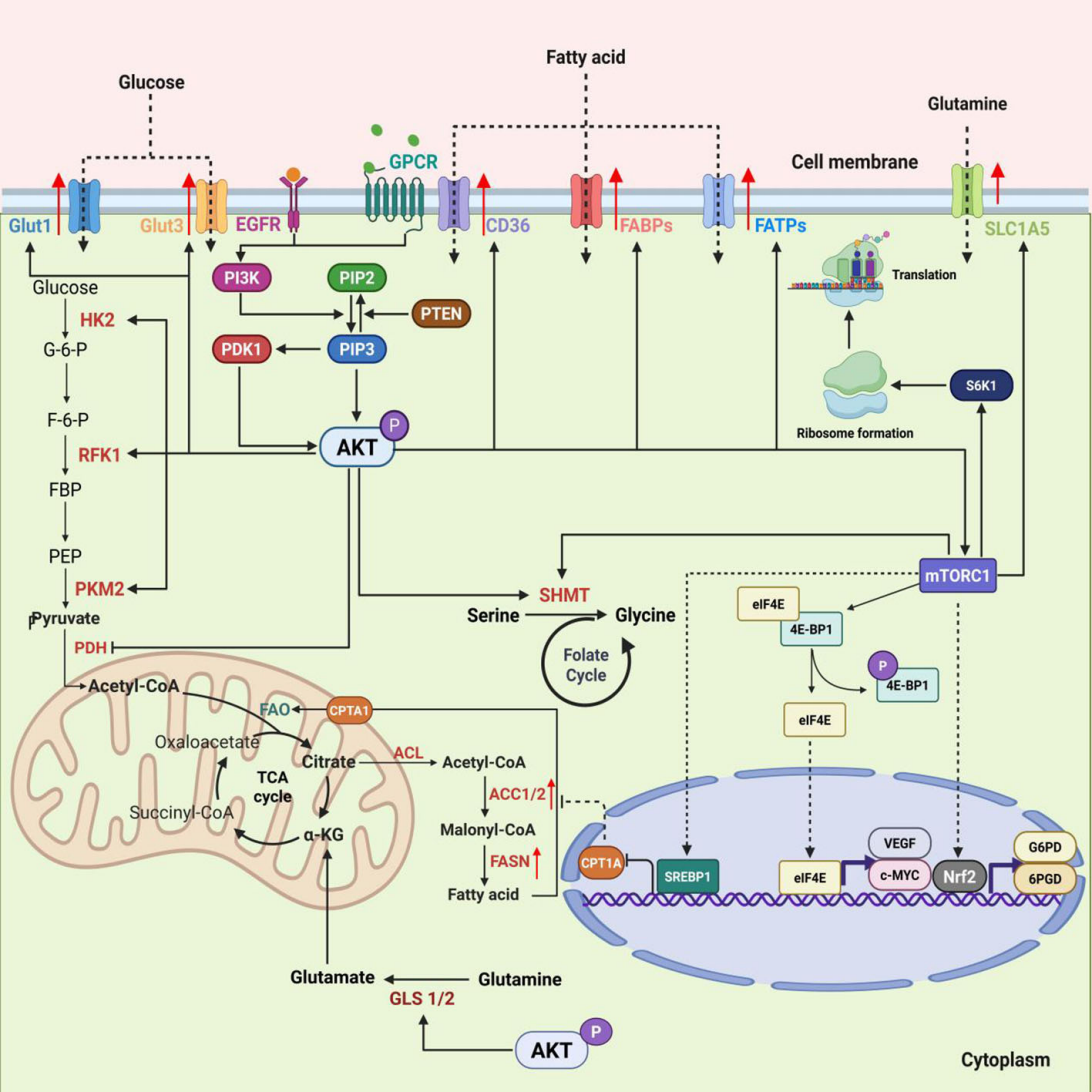
Core mechanisms of AKT/mTORC1 signaling axis in regulating tumor metabolic reprogramming. AKT (protein kinase B) receives upstream signals from EGFR (epidermal growth factor receptor), GPCR (G protein-coupled receptor), and other receptors via the PI3K/AKT pathway, and collaborates with mTORC1 (mechanistic target of rapamycin complex 1) to orchestrate glucose, fatty acid, and glutamine metabolic reprogramming. AKT promotes glucose uptake and glycolysis (GLUT1/3, HK2, PKM2). The AKT/mTORC1-SREBP1 pathway stimulates fatty acid synthesis (ACC1/2, FASN) and uptake (CD36, FATPs), while inhibiting CPT1A-dependent FAO. AKT enhances glutamine uptake and breakdown (SLC1A5, GLS1/2) to feed the TCA cycle. mTORC1 promotes protein translation via 4E-binding protein 1 (4E-BP1) and ribosomal protein S6 kinase 1 (S6K1), and amplifies reprogramming through MYC and nuclear factor erythroid 2-related factor 2(Nrf2). The PI3K-AKT-mTORC1 axis is a central regulator that integrates growth signals to drive glycolysis, glutamine catabolism, and lipogenesis, while suppressing FAO. It enables the anabolic state required for rapid tumor growth and drug resistance.

In terms of fatty acid metabolism,mTORC1 plays a crucial role by activating sterol regulatory element-binding proteins (SREBPs) (100, 101). SREBPs are transcription factors that, when activated within the cell nucleus, increase the expression of genes required for lipid biosynthesis, such as acetyl-CoA carboxylase (ACC) and fatty acid synthase (FAS) (102). This process is necessary for the creation of cholesterol and fatty acids. SREBPs have the ability to upregulate fatty acid transporters such as CD36, FABPs, and FATPs. Furthermore, SREBPs can reduce the production of carnitine palmitoyltransferase 1A (CPT1A), which prevents fatty acid translocation into mitochondria and inhibits fatty acid oxidation. This inhibition guarantees that tumor cells minimize lipid catabolism while synthesizing lipids, which promotes additional lipid buildup. Abnormal activation of the mTORC1-SREBP pathway in illnesses such as cancer, obesity, and hepatic steatosis causes lipid metabolism problems (**Figure 3**).

## 4 Tumor metabolic reprogramming and its interaction with the tumor microenvironment

**Figure 4:**
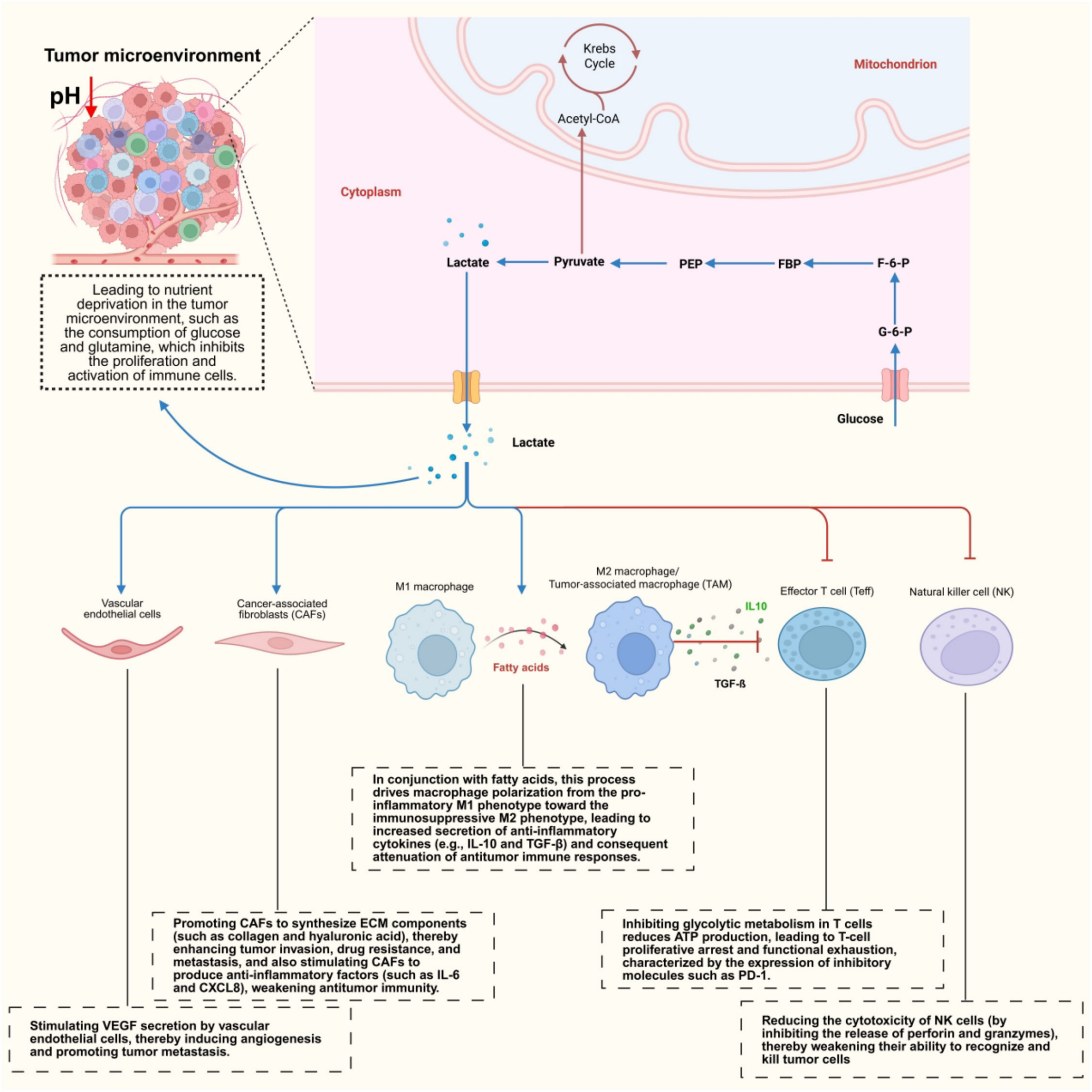
Metabolic reprogramming and tumor microenvironment interactions. This figure depicts the bidirectional metabolic crosstalk between tumor cells and the tumor microenvironment (TME). Lactate accumulation, nutrient competition, and hypoxia drive immune suppression, M2 macrophage polarization, CAF activation, angiogenesis, and immune escape.

The tumor micro-environment (TME) is a complex dynamic system made up of cellular and non-cellular components such as tumor cells, stromal cells, immune cells, extracellular matrix (ECM), and a variety of signaling chemicals (**Figure 4**) (103). Metabolic interactions within the tumor microenvironment are not unidirectional; rather, they form complex, bidirectional, symbiotic relationships centered on tumor cells and involving surrounding stromal cells (such as fibroblasts), endothelial cells, and immune cells. The aberrant metabolic activity of tumor cells has a major impact on the makeup and function of the tumor microenvironment. This milieu encourages tumor growth, invasion, and metastasis, resulting in a vicious cycle.

The hypoxic microenvironment created by fast tumor cell proliferation is a primary cause of metabolic reprogramming. HIF-1α, a critical regulatory factor, promotes the transcription of genes like GLUT1 and LDHA, increasing glycolysis in tumor cells (25). Simultaneously, it inhibits the production of mitochondrial respiratory chain components. Hypoxia also induces M2 polarization in TAMs (104). M2 macrophages often maintain their immunosuppressive phenotype by increasing fatty acid oxidation (FAO), which promotes tumorigenesis and progression (105). Under hypoxic conditions, TAMs undergo metabolic reprogramming, which promotes tumor development and immune evasion. Furthermore, intermittent hypoxia (IH) has been shown to increase TAM-induced glycolysis in tumor cells, which is similarly linked to M2 macrophage-driven tumor invasiveness (106). This phenomenon reveals that even intermittent hypoxic environments can promote tumor development by altering TAM metabolism.

Lactate causes acidification of the TME. The pH can drop to 6.0–6.5, significantly lower than that of normal tissue (107). This acidic environment suppresses the function of immune cells, such as the activity of cytotoxic T lymphocytes (CTLs) and natural killer (NK) cells, thereby enabling immune evasion (108). Lactic acid acts as a signaling molecule, regulating gene expression in tumor cells and TAMs. It promotes the secretion of immunosuppressive cytokines such as interleukin-10 (IL-10) and transforming growth factor-β (TGF-β). Lactic acid can also be reutilized by tumor cells via MCT1/4 transporters, and it also functions as a signaling molecule, activating GPR81 receptors and boosting VEGF secretion to stimulate angiogenesis. While this acidic environment may promote tumor cell invasion and immune evasion, it also causes stress on the tumor cells, reducing enzyme activity and cellular function. Catalase (CAT) catalyzes O_2_ synthesis in tumor tissues, which helps alleviate hypoxia. However, the acidic tumor microenvironment dramatically lowers CAT activity, reducing its efficacy in treating hypoxia. This method reveals the potential metabolic sensitivity of the tumor microenvironment, providing a new therapeutic target for cancer treatment.

In addition to receptor-mediated signaling and microenvironmental acidification, lactic acid-driven immunological actions comprise a complex regulation mechanism based on direct molecular alteration. Tumor immune suppression is largely mediated by direct action (histone lactylation), however the predominant mechanisms differ among immune cell types. By directly covalently binding to histone lysine residues (like H3K18 and H4K5) without the need for receptor or acidification mediation, lactate can change chromatin accessibility as a direct substrate for histone lysine lactylation (Kla), an epigenetic modification that controls the transcription of immune-related genes (109). This is the fundamental mechanism underlying its immunological effects. Direct lactate supplementation has been shown *in vitro* to dose-dependently promote histone lactylation and the expression of PD-L1 and M2 macrophage markers. Additionally, this impact is not affected by changes in extracellular pH; it is diminished when the lactate transporter MCT1 is knocked down but reinstated when lactate is delivered intracellularly. The dominant mechanisms of various immune cell subsets differ significantly: in CD8+ T cells, the main mechanisms are PD-L1 upregulation mediated by histone lactylation and competitive inhibition of glycolytic enzymes by lactate, with microenvironmental acidification acting as a secondary factor; in TAMs, histone lactylation and receptor-mediated actions work in concert, with acidification helping to stabilize the M2 phenotype (110); in DCs, microenvironmental acidification is the primary mechanism, with no evidence of histone lactylation; and in AML tumor cells, histone lactylation-mediated upregulation of PD-L1. In conclusion, immunotherapy that targets lactate metabolism needs to choose specific targets according to the kind of tumor and immune cell subpopulation.

The interaction with PD-L1 on tumor cells and PD-1 on T cells might cause T cell exhaustion. Tumor cells promote PD-L1 expression through a variety of methods. In hyperglycemic conditions, HK2 causes IκBα phosphorylation, degradation, and upregulation of PD-L1 expression, ultimately suppressing T cell function (111). This inhibition is accomplished by engaging protein tyrosine phosphatase 2 (SHP2), which dephosphorylates critical signaling molecules in T cell activation pathways like the PI3K-AKT pathway (112). This inhibits the activation of transcription factors including NFAT, NF-κB, and BATF, which regulate gene expression for T cell activation, proliferation, and effector activities. Ultimately, this causes T cell depletion and increases tumor immune escape. In recent years, immune checkpoint drugs that target the PD-1/PD-L1 pathway, such as anti-PD-1 antibodies and anti-PD-L1 antibodies, have made major advances in cancer treatment. These medicines revive fatigued T cells by inhibiting PD-1 binding to PD-L1, restoring antitumor activity.

The metabolic activity of tumor cells also leads to nutrient depletion in TME, such as the consumption of glucose, glutamine, and other substances (113). Deficiencies in nutrients impair the metabolism and function of immune cells, placing them in a state of stress and inhibiting their activation and proliferation (114). For example, although T cells possess a certain degree of metabolic plasticity, their proliferation capacity, cell cycle progression, and ability to secrete cytokines are significantly impaired in glucose-deprived environments, rendering them unable to effectively exert antitumor effects (115). Additionally, tumor cells secrete chemokines and growth factors that recruit and activate cells such as CAFs and TAMs (116). These cells further secrete extracellular matrix and cytokines, promoting tumor angiogenesis and tumor cell migration, and influencing tumor cells’ sensitivity to anticancer drugs (117). Regulatory T cells maintain their suppressive function by enhancing lipid oxidation and lactate metabolism adaptation (e.g., the CD38-NAD^+^ metabolic axis); Myeloid suppressor cells expand under VISTA/polyamine signaling and release metabolites such as methylglyoxal to directly paralyze T cells; dendritic cells experience impaired FLCN-TFEB signaling due to failed competition for the glutamine transporter SLC38A2, leading to compromised antigen presentation. Together, these mechanisms form a complex metabolic immune checkpoint network, providing a theoretical basis for the combined targeting of metabolic pathways to reverse immune suppression.

Metabolic coupling between tumor cells, CAFs, and TAMs is an extremely complicated type of intercellular symbiosis. This metabolic crosstalk is a bidirectional dialogue formed by oncogenic signaling, hypoxia, acidification, and inflammatory signals in TME.A prototypical example of such linkage is the “reverse Warburg effect,” which was first postulated by Lisanti and colleagues and has transformed the conventional view of tumor metabolism. In this paradigm, CAFs conduct aerobic glycolysis in response to oxidative stress and paracrine signals generated by nearby cancer cells, resulting in high levels of lactate and pyruvate. These compounds are then exported via monocarboxylate transporter 4 (MCT4) and absorbed by cancer cells via MCT1, where they act as oxidative fuels in mitochondria, supporting ATP generation and metabolic pathways.

The extracellular matrix (ECM), which serves as the structural underpinning for TME, governs metabolic processes by controlling both physical-mechanical and chemical signals. The ECM promotes integrin signaling pathways, which, via focal adhesion kinase (FAK) and Src, activate downstream pathways such as the PI3K-AKT-mTOR pathway, hence increasing tumor cell motility and invasion. Excess collagen fiber deposition stiffens the tissue, forming a physical barrier to anticancer drug delivery and restricting drug distribution within tumors (118). Hyaluronic acid breakdown products improve tumor cells’ ability to adapt to hypoxic conditions by stabilizing HIF-1α. The ECM can also create regionally diverse nutritional gradients by binding nutrients like glucose and glutamine, causing metabolic phenotypic differentiation in tumor cells across areas.

Achieving complete normalization of the tumor microenvironment is a major challenge, as tumor metabolic reprogramming is multifaceted and highly adaptive; however, combining metabolic targeted therapies with immune checkpoint inhibitors (ICIs) or other immunotherapies may normalize the tumor microenvironment and enhance treatment efficacy.

There are numerous information gaps and unanswered scientific concerns in the study of tumor cell interactions with the tumor microenvironment. For example, tumor cells and immunological cells struggle for nutrients. Under what conditions does this competitive metabolic model succeed? Does this competition fluctuate at different phases of tumor progression? The majority of available studies are conducted under static *in vitro* circumstances, and there is a dearth of spatiotemporal resolution data on dynamic changes in metabolic interactions in the microenvironment. Furthermore, the revelation that gut microbiota metabolites might modify the tumor’s nutritional microenvironment has broadened the scope of tumor metabolic reprogramming studies. However, research in this area is still mostly descriptive; the specific molecular processes and causal pathways via which microbiota-host metabolic interactions drive tumor metabolic reprogramming have yet to be identified. There is a dearth of systematic information as to whether microbiome components or metabolites play important roles in specific tumor forms.

## 5 Clinical translation of tumor metabolism

### 5.1 Diagnostic methods based on tumor metabolism

Metabolic abnormalities in tumor cells offer new insights and approaches for cancer diagnosis.

Positron Emission Tomography (PET) is a clinically used diagnostic tool that relies on tumor metabolism. Its principle is based on tumor cells’ high glucose absorption. FDG uptake into tumor tissue is detected by injecting the radioactively tagged glucose analog fluorodeoxyglucose (FDG), allowing for tumor localization and identification (119). PET is used to diagnose, stage, and assess treatment response for a variety of malignancies. For example, in non-small cell lung cancer, it is utilized for lesion detection, diagnosis and differential diagnosis, pathological staging, assessment of therapy efficacy and prognosis, recurrence detection, and metastasis detection (120). However, PET has certain drawbacks, including decreased sensitivity in detecting some low-metabolic cancers and the inability to offer precise metabolic information about tumor cells (121). For example, in prostate cancer, energy supply is primarily reliant on citrate oxidation. PET scans based on glucose uptake cannot accurately distinguish between healthy and malignant prostate tissue, necessitating the use of alternative diagnostic procedures such as magnetic resonance spectroscopy (122).

The improvement of metabolomics technology also allows for more extensive and in-depth methods to tumor diagnostics. Metabolomics can detect small changes in tumor cell metabolism and find potential tumor biomarkers by studying the metabolite profiles in biological samples. Metabolomic analysis of blood, urine, or tissue samples from cancer patients reveals metabolites linked to tumor development and progression (123). Spatial metabolomics allows for direct *in situ* mapping of metabolite distribution profiles while preserving critical histology information about malignancies. This demonstrates that the tumor metabolic phenotype is not uniformly distributed, but rather creates complicated and dynamic gradients that are tightly linked to histological regions, vascular proximity, hypoxia levels, and immune cell infiltration patterns. Lactate, a hallmark of aerobic glycolysis, is predominantly enriched in hypoxic tumor cores at concentrations of 10–40 mM, according to studies using platforms such as matrix-assisted laser desorption/ionization mass spectrometry imaging (MALDI-MSI) and desorption electrospray ionization mass spectrometry imaging (DESI-MSI), with markedly reduced glucose and oxygen levels in corresponding regions. This distinct metabolic milieu created by spatial compartmentalization directly influences cell behavior and treatment responsiveness. The research value of this technology extends beyond the description of metabolite distribution; it also provides functional insights into tumor-immune crosstalk.

### 5.2 Therapeutic strategies based on tumor metabolism

One of the major hotspots in cancer therapy research is the development of specialized metabolic inhibitors that target tumor cell metabolic abnormalities (**Table 1**). Many metabolic enzymes and transporters have emerged as prospective therapeutic targets, with relevant metabolic inhibitors now in clinical studies or approved for market release. Mutations in IDH1 and IDH2 are common in a variety of malignancies, including acute myeloid leukemia and gliomas (124). Mutated IDH enzymes generate the aberrant metabolic byproduct 2-HG, which stimulates tumor cell proliferation and survival (125). IDH inhibitors specifically block the action of mutant IDH enzymes, lowering 2-HG levels while causing tumor cell differentiation and apoptosis. Multiple IDH inhibitors are currently approved for the treatment of patients with IDH-mutated malignancies (126). Clinical investigations have shown that IDH inhibitors increase survival and enhance patient outcomes.

**Table 1:** Major tumor metabolic inhibitors.

Drug	Target	Indication	Clinical phase	Key findings and limitations
Phenformin	Mitochondrial complex I	Pancreatic ductal adenocarcinoma (PDAC), etc.	Preclinical/Early clinical exploration	Superior efficacy to multiple metabolic inhibitors observed in PDAC patient-derived xenograt (PDX) models
Telaglenastat (CB-839)	Glutaminase (GLS)	Renal cell carcinoma, breast cancer, etc.	Phase I–II	*In vivo* translation challenged by redundancy in glutamine metabolism
6-Diazo-5-oxo-L-norleucine (DON)	Broad-spectrum glutamine antagonist	Various cancers	Phase I–II	Phase I/II trials terminated due to dose-limiting nausea and vomiting
2-Deoxy-D-glucose (2-DG)	Hexokinase/Glycolysis	Various solid tumors	Phase I–II	Hindered in clinical trials by systemic toxicity and limited efficacy
Denifanstat (TVB-2640)	Fatty acid synthase (FASN)	Solid tumors, NASH	Phase I–II	FASN inhibitor; representative agent for targeted lipid metabolism research
Antifolates (Methotrexate/Pemetrexed, etc.)	DHFR/TS/One-carbon metabolism	Leukemia, NSCLC, mesothelioma, etc.	Approved	Earliest class of metabolic inhibitors.
Ivosidenib (AG-120), Enasidenib (AG-221)	Mutant IDH1/IDH2	AML	FDA-approved	Precisely blocks production of the oncometabolite 2-HG; represents a successful paradigm of precision medicine in targeted metabolism therapy

Glutaminase inhibitors have received a lot of attention in tumor therapy research. Inhibiting glutaminase prevents glutamine metabolism, depriving tumor cells of an important nutrient supply and slowing tumor growth (127). Glutaminase inhibitors increase tumor cell radiosensitivity by depleting glutathione and enhancing radiation-induced DNA damage, such as in KEAP1-mutated lung adenocarcinoma cells (128). Targeting glutamine metabolism enhances the tumor immunological microenvironment, which boosts anticancer immune responses (129). During clinical trials, CB-839 has advanced into multiple Phase I/II studies, with attempts to combine it with chemotherapy, targeted therapy, and immune checkpoint inhibitors (130). In a Phase Ib/II clinical trial (NCT02071862) of patients with advanced TNBC, CB-839 in combination with paclitaxel improved disease control but did not significantly improve objective response rate (ORR) or progression-free survival (PFS) in subsequent large-scale studies.

Furthermore, great progress has been achieved in tumor treatment studies using inhibitors such as fatty acid synthase and glucose transporter inhibitors (131).

However, metabolic compartmentalization within tumors refers to the formation of specific metabolic microenvironments within TME between different cell subpopulations—such as cancer cells, surrounding stromal cells, and immune cells—as well as among different subcellular structures within the same cell (e.g., mitochondria, lysosomes, and peroxisomes). This leads to significant heterogeneity in substrate utilization, metabolic flux, and redox status. For example, mitochondria play a central role in the metabolic reprogramming of cancer cells. Many cancer cells exhibit mitochondrial dysfunction yet are able to meet their proliferative demands through mitochondrial metabolism. Lysosomes perform critical functions in nutrient sensing, metabolite recycling, and drug degradation; their acidic environment may mediate the inactivation of certain drugs, thereby affecting therapeutic outcomes. This complex metabolic landscape profoundly influences tumor progression, treatment response, and drug resistance, presenting new challenges to traditional therapeutic targeting strategies.

The effectiveness of tumor treatment can be increased by combining metabolic inhibitors with conventional chemotherapy, radiation, or immunotherapy (132). For instance, glycolysis inhibitors can increase the deadly effects of chemotherapy by preventing tumor cells from receiving energy (133). In the field of immunotherapy, metabolic inhibitors can improve the tumor micro-environment, enhance the function of immune cells, and increase the efficacy of immunotherapy (134).

## 6 Limitations in targeting tumor metabolism

Three major critical challenges remain in current therapeutic strategies, which necessitate in-depth study.

First, tumor cell metabolism is highly heterogeneous. Tumor cell metabolic patterns are not consistent; they differ greatly across tumor types, places of origin, development stages, and microenvironments. Because of this variability, single metabolic targeting cannot cover all tumor cell subpopulations. This not only diminishes treatment efficacy but also rapidly promotes drug resistance in residual tumor cells, raising the chance of tumor recurrence. This is the primary hurdle in the clinical use of metabolic focused treatments. Additionally, the universality of metabolic pathways can result in medication toxicity. Many metabolic targets are also vital in normal cells, therefore blocking tumor metabolism can readily disrupt normal cellular functioning. Glutamine and glycolysis inhibitors, for example, have the potential to harm normal tissues such as red blood cells. To limit toxicity, drug development must take into account the features of both tumor and non-tumor cells, specifically specifying the impact of metabolic inhibitors on normal cells. Finally, the complicated mechanisms governing interactions between tumor metabolism and other cells in the tumor microenvironment (e.g., immune cells, fibroblasts) are still poorly understood, restricting future development of combination therapies targeting tumor metabolism. Immune cells, fibroblasts, and other cells in the tumor microenvironment form complex interactions with tumor cells. These interactions not only affect tumor metabolic reprogramming, but also immune evasion, invasion, and metastasis. The uncertainty of these processes restricts the development and implementation of tumor metabolism-based combination treatment regimens.

Inhibitors that target important enzymes like CPT1 or FASN have demonstrated anticancer effect in preclinical models, but comparatively few successful instances have been documented in clinical trials. This is a noteworthy example of a failure. Many medications were stopped early on because of problems with toxicity or inadequate efficacy.

Drug safety must be considered while guaranteeing medication efficacy. This can be divided into three aspects: drug design optimization, adjustment of therapeutic strategies, and management of toxicity. First, taking advantage of the fact that the mitochondrial membrane potential of tumor cells is significantly higher than that of normal cells, the drug is conjugated with the lipophilic cation triphenylphosphine (TPP+), enabling it to selectively accumulate at high concentrations in tumor mitochondria. Second, moderate-potency inhibitors are employed: while potent inhibitors demonstrate significant efficacy, they often have an extremely narrow therapeutic window and are prone to causing severe toxicity. For example, the potent oxidative phosphorylation (OXPHOS) complex I inhibitor IACS-010759 had its clinical trials terminated due to dose-limiting toxicities (such as lactic acidosis and neuropathy). In contrast, some milder OXPHOS inhibitors, such as the antimalarial drug atovaquone and the antispasmodic papaverine, can moderately inhibit tumor oxygen consumption and improve tumor hypoxia at clinically achievable concentrations, thereby enhancing the efficacy of radiotherapy while maintaining good safety profiles. Third, for drugs that cause specific, predictable metabolic toxicity due to their effects on key signaling pathways, proactive monitoring and management are crucial to ensuring the treatment proceeds smoothly. Take inhibitors of the PI3K-Akt-mTOR (PAM) pathway as an example; these drugs often cause hyperglycemia and hyperlipidemia. Expert consensus recommends that routine screening and monitoring for these metabolic abnormalities be conducted during clinical trials and clinical practice, with timely intervention (such as the use of antidiabetic or lipid-lowering medications). In most cases, these toxicities are manageable and reversible; therefore, dose reduction or discontinuation should not be initiated immediately. Adjustments to the treatment regimen should only be considered in cases of severe or persistent deterioration.

## 7 Future perspectives

To overcome the limitations and challenges of tumor metabolism, future research in this field should focus on core issues and promote breakthroughs along three dimensions.

At the mechanistic level, it is critical to investigate the molecular regulatory mechanisms that underpin tumor metabolic heterogeneity, identify the key drivers of metabolic reprogramming across tumor subtypes and developmental stages, and lay the theoretical groundwork for personalized metabolic targeted therapies.

In terms of technical innovation, developing and implementing innovative metabolic detection techniques are essential facilitators. AI-driven metabolomics, single-cell metabolomics, and spatial metabolomics technologies can dynamically and precisely capture the spatiotemporal characteristics of tumor metabolic states, providing quantitative markers for tumor subtyping, treatment efficacy assessment, and drug resistance prediction. Metabolomics maps the metabolic landscape of cancer by quantifying metabolite levels in tissue and body fluid samples, and the use of AI transforms this data into therapeutically usable precision oncology tools. The primary benefit of AI in tumor metabolism research is not only the processing of high-dimensional data, but also the discovery of complex interdependencies across several omics levels. AI’s comprehensive application in cancer drug resistance research includes six key dimensions: drug development, resistance mechanism elucidation, drug sensitivity prediction, combination therapy optimization, resistance phenotype identification, and clinical biomarker discovery. For metabolic inhibitors, AI’s capacity to forecast medication resistance is especially important. The breakthrough significance of spatial metabolomics resides in its ability to answer a fundamental topic that traditional omics techniques cannot: the heterogeneous distribution of metabolic reprogramming inside tissue areas and its relevance to intercellular interactions. The technology landscape for spatial transcriptomics and proteomics is quickly evolving. Core technologies are divided into two categories: sequencing-based capture methods (e.g., Visium) and imaging-based *in situ* detection methods (e.g., IMC, MERFISH), which have intrinsic trade-offs in resolution, throughput, and coverage. It is important to note that spatial biomarkers include information about the spatial arrangement of molecules, and their predictive power far exceeds that of classic non-spatial biomarkers, indicating enormous potential, particularly in tumor immunotherapy and tailored treatment guiding. Single-cell multi-omics can reveal the molecular underpinnings of tumor heterogeneity and metabolic flexibility at unprecedented detail.

Simultaneously, the combined use of multi-omics technologies—deeply integrating metabolomics with genomes, transcriptomics, and proteomics—can uncover the intricate regulatory networks that drive tumor metabolism. At the therapeutic strategy level, combination therapy will become the standard technique in cancer treatment. By combining metabolic inhibitors with traditional modalities such as chemotherapy, radiation, and immunotherapy, this method efficiently overcomes drug resistance caused by tumor metabolic heterogeneity while lowering treatment toxicity. This boosts anticancer efficacy and increases long-term patient survival rates.

## 8 Discussion

Tumor metabolism, as a core subject in cancer research, provides important theoretical foundations and research prospects for understanding tumor features and generating novel therapeutic methods. This article provides a systematic assessment of the key elements of metabolic reprogramming in cancer cells, including molecular regulatory mechanisms, interaction networks within the tumor microenvironment, and clinical translation progress. It clarifies the critical roles of multiple metabolic pathways in tumor proliferation, invasion, drug resistance, and immune evasion, as well as the global regulation of metabolic networks by driver genes like MYC and p53 and signaling pathways like PI3K/AKT/mTOR, providing a theoretical foundation for tumor-specific precision therapy.

Compared to similar literature, this study provides a more systematic and thorough summary. It addresses the limits of prior reviews, which were largely concerned with glucose, amino acid, and fatty acid metabolism, by introducing cutting-edge issues like as one-carbon metabolism, ferroptosis, and cuptosis, thereby improving the metabolic network. Furthermore, it examines the bidirectional relationships between metabolism, the tumor microenvironment, and immune suppression, revealing new mechanisms such as the reverse Warburg effect and questioning the single-dimensional story of metabolism. Finally, this review focuses on the clinical bottlenecks and causes for failure associated with metabolism-targeted medicines. It paves the way for future research by combining AI, spatial omics, and single-cell metabolomics in precision diagnosis and treatment.

To summarize, tumor metabolism research has progressed from the investigation of fundamental pathways to a vital stage of clinical translation toward precision medicine. Targeting tumor metabolism will provide a new paradigm for precision cancer therapy, significantly improving patient outcomes, enhancing clinical diagnostic and therapeutic standards, and propelling cancer treatment into a new era of precision medicine, as our understanding of these mechanisms continues to deepen, detection technologies advance iteratively, and combination therapy regimens are continuously optimized.
